# Correlation between neutrophil extracellular traps and macrophages in thrombi of patients with acute ischemic stroke

**DOI:** 10.1371/journal.pone.0329764

**Published:** 2025-08-26

**Authors:** Mingming Zang, Ruodong Han, Xiuxia Yan

**Affiliations:** The Affiliated Bozhou Hospital of Anhui Medical University, Bozhou, China; UCSF: University of California San Francisco, UNITED STATES OF AMERICA

## Abstract

**Objective:**

To investigate the correlation between Neutrophil Extracellular Traps (NETs) content and macrophages in thrombi of acute ischemic stroke (AIS) patients, as well as the differential degradation and clearance capacities of macrophages polarized into distinct functional states.

**Methods:**

60 AIS patients treated with endovascular mechanical thrombectomy at Bozhou People’s Hospital were enrolled. Thrombus samples from 30 patients underwent immunohistochemical staining for citrullinated histone 3 (CitH3), CD16, and CD163. CitH3-positive area percentage was quantified to evaluate NETs content. Pearson’s correlation analysis was applied to assess associations between M1(CD16⁺) and M2(CD163⁺) macrophage densities and the CitH3-positive area in thrombus. For the remaining 30 thrombi, co-culture experiments with polarized macrophages were conducted. CitH3 concentrations before and after co-culture were measured via enzyme-linked immunosorbent assay (ELISA), with a blank control group as a reference. Statistical comparisons between groups were performed using Student’s t-tests.

**Results:**

All 30 thrombi exhibited positive expression of CitH3, CD16, and CD163. CD16^+^ and CD163^+^ macrophage densities significantly correlated with CitH3-positive area percentage (r = 0.538 and 0.641, *P* < 0.05). Co-culture with M1 or M2 macrophages significantly reduced CitH3 concentrations compared to the blank control (*P* < 0.05). Notably, M1 macrophages demonstrated superior NETs degradation efficacy compared to M2, as evidenced by lower post-co-culture CitH3 levels (*P* = 0.038).

**Conclusion:**

NETs are contained in the thrombus of patients with acute ischemic stroke. The numbers of M1 macrophages and M2 macrophages in thrombus are positively correlated with the content of NETs. M1 and M2 macrophages derived from human monocytes have the ability to degrade and clear NETs, and the effect of M1 macrophages in degrading and clearing NETs may be stronger than M2 macrophages.

## Introduction

Stroke, characterized by high incidence, disability, mortality, and recurrence rates, imposes a profound socioeconomic burden globally. Ischemic stroke, the most prevalent subtype, accounts for 69.6–72.8% of newly diagnosed stroke cases in China [1,2]. Current therapeutic strategies for acute ischemic stroke (AIS)—primarily endovascular mechanical thrombectomy and thrombolysis—aim to rapidly restore cerebral perfusion, mitigate ischemic-hypoxic injury, and reduce infarct core expansion. However, stringent eligibility criteria and contraindications limit their applicability, leaving a subset of patients without effective treatment and at risk of poor outcomes. Consequently, there is an urgent clinical need for novel therapeutic approaches to improve AIS management.

Neutrophil extracellular traps (NETs), extracellular DNA scaffolds released by activated neutrophils, were first described by Volker Brinkmann et al. as a novel antimicrobial mechanism [3]. Emerging evidence has highlighted their critical roles in the pathogenesis of ischemic stroke [4–7]. After AIS, damaged endothelial cells and neurons release chemokines and cytokines, which activate Toll-like receptors and trigger inflammatory cascades [4]. Recruited neutrophils exacerbate neuroinflammation by releasing pro-inflammatory mediators and NETs, which in turn amplify cytokine production, establishing a feedforward loop that worsens ischemic injury [5–7]. Structurally, NETs act as a scaffold for thrombus formation, promoting platelet adhesion, activation, and aggregation, while stabilizing clots through interactions with fibronectin, fibrinogen, and von Willebrand factor (vWF) [8]. Notably, Julia Novotny et al. demonstrated that NETs content in cerebral thrombi correlates with clinical prognosis, underscoring their pathophysiological significance [9]. Citrullinated histone 3 (CitH3), a hallmark of NETs formation mediated by peptidylarginine deiminase 4 (PAD4), serves as a reliable biomarker for NETs detection via immunohistochemistry, immunofluorescence, or ELISA [10].

Macrophages, tissue-resident immune cells derived from monocyte differentiation, exhibit functional plasticity shaped by microenvironmental cues. Classically polarized into pro-inflammatory M1 or anti-inflammatory M2 subtypes, macrophages express distinct surface markers (e.g., CD16, CD80/86 for M1; CD163, CD206 for M2) and regulate diverse immune responses. In vitro studies suggest that macrophages recognize extracellular DNA and phagocytose NETs [11,12]. Furthermore, Manda-Handzlik et al. reported that secretions from both M1 and M2 macrophages suppress NETs release by neutrophils, implicating paracrine regulation in NETs dynamics [13]. Despite these advances, the interplay between thrombi-resident NETs and macrophages in AIS remains poorly understood, particularly regarding macrophage-mediated NETs clearance. Given the significant role of neutrophils and NETs in ischemic stroke pathogenesis, exploring the correlation between NETs and macrophages, as well as macrophages’ capacities to degrade and clear NETs, becomes highly relevant and crucial for developing novel therapeutic strategies against AIS.

## Materials and methods

### Study population

A total of 60 patients with AIS who underwent endovascular mechanical thrombectomy at Bozhou People’s Hospital between 1 December 2022 and 31 December 2024 were consecutively enrolled in this study. Written informed consent was obtained from all participants or their legally authorized representatives prior to enrollment. Thrombus samples were collected during the procedure, with 30 samples allocated for immunohistochemical analysis and the remaining 30 for macrophage co-culture experiments. Peripheral blood (15 mL) was drawn preoperatively from patients undergoing co-culture experiments for macrophage isolation. The study protocol was approved by the Ethics Committee of Anhui Medical University (Approval No. 83220469).

Inclusion Criteria were as follows: (1)Diagnosis of AIS according to the 2018 Chinese Guidelines for the Diagnosis and Treatment of AIS [14]; (2)Patients with ischemic stroke classified as large-artery atherosclerosis with cerebral thrombosis according to the TOAST classification; (3)Patients undergoing endovascular mechanical thrombectomy, regardless of whether thrombolysis was performed prior to thrombectomy.; (4)Age range: 18–90 years.

Exclusion Criteria were as follows: (1)AIS etiology is classified as cardioembolic, small-vessel occlusion, other determined causes, or undetermined causes; (2)Patients not undergoing endovascular mechanical thrombectomy; (3)Severe multi-organ dysfunction; (4)Comorbid immune disorders.

### Study design and procedures

30 brain thrombus samples were fixed with 4% paraformaldehyde, then dehydrated, cleared, infiltrated, embedded and sectioned. Immunohistochemical staining for CitH3, CD16 and CD163 was performed on the paraffin sections. ImageJ (version:1.53c, National Institutes of Health, USA) was used to analyze the images. Since NETs in thrombi have a mesh structure and can’t be quantified, CitH3-positive area in thrombi was measured, and the percentage of CitH3-positive area to thrombus section area was used to reflect NETs content. For M1 and M2 macrophages, CD16^+^ and CD163^+^ macrophages in thrombi were counted, and their numbers per unit area (mm^2^) were used to reflect their quantities.30 thrombus samples used for co-culture were ground into homogenate with a sample cryo-grinder under sterile conditions. The homogenate was centrifuged at 5000 r/min for 10 minutes, and the supernatant was collected into a sterile EP tube and stored in a −20°C refrigerator for further use.

### Macrophage isolation, polarization, and co-culture

Prior to mechanical thrombectomy, 15 mL of peripheral blood was collected from patients undergoing thrombus-macrophage co-culture. EDTA was added as an anticoagulant, and peripheral blood mononuclear cells (PBMCs) were isolated using Ficoll density gradient centrifugation. The obtained PBMCs were seeded into culture dishes and incubated in an appropriate volume of RPMI-1640 complete medium for 2 hours. After incubation, the medium was replaced to remove non-adherent lymphocytes, which grow in suspension. The adherent monocytes (which typically adhere within 2–4 hours) were then harvested using trypsin digestion, centrifuged, and resuspended in RPMI-1640 complete medium containing 100 ng/mL macrophage colony-stimulating factor (M-CSF). The cell suspension was adjusted to a concentration of 5 × 10⁵ cells/mL.

A total of 6 mL of the prepared cell suspension was divided into two 25 cm^2^ culture flasks, with 3 mL added to each flask, and incubated at 37°C in a CO₂ incubator. Additionally, 2 mL of the suspension was added to a 24-well culture plate, with 0.5 mL per well (4 wells in total), and the volume was adjusted to 1 mL per well with the same medium for synchronized cell culture. The cells were cultured for 6 days, with the medium changed every 2 days.On the sixth day, the culture medium in the two flasks was replaced with 3 mL of RPMI-1640 complete medium containing either 100 ng/mL M-CSF, 100 ng/mL lipopolysaccharide (LPS), and 20 ng/mL interferon-γ (IFN-γ) to induce M1 macrophage polarization, or 100 ng/mL M-CSF and 20 ng/mL interleukin-6 (IL-6) to induce M2 macrophage polarization. The cells were incubated for 18 hours. In the 24-well plate, two wells were treated with each of these polarization media for subsequent culture. After polarization, CD16 and CD163 fluorescence staining was performed on the cells in the 24-well plate to confirm M1 or M2 macrophage differentiation.

Following identification, 1 mL of thrombus homogenate supernatant was mixed with 9 mL of RPMI-1640 complete medium, and 1 mL was stored at −20°C for pre-culture CitH3 concentration analysis. Then, 3 mL of the thrombus homogenate-containing medium was added to each flask containing M1 or M2 macrophages for co-culture. A blank control group was set up by adding 3 mL of the same medium to an empty culture flask. The cells were incubated at 37°C in a CO₂ incubator for 48 hours.After co-culture, the culture supernatants from both the experimental and control groups were collected and centrifuged at 5000 r/min for 10 minutes. The supernatants were transferred into EP tubes and stored at −20°C for further analysis.

### NETs quantification

CitH3 is a specific marker of NETs formation; therefore, the concentration of CitH3 in the culture medium before and after co-culture was measured to assess changes in NETs levels. A series of standard solutions were prepared according to the instructions of the human CitH3 ELISA kit. The absorbance values of the standards at 450 nm were measured using a microplate reader, and a standard regression curve was plotted. The absorbance values of the culture medium before co-culture, after thrombus-M1 or M2 macrophage co-culture, and from the blank control group were then measured at 450 nm. The CitH3 concentration was calculated based on the standard curve.

### Statistical analysis

Continuous variables are presented as mean (SD). Between-group comparisons were performed using independent samples t-tests. Pearson’s correlation analysis was used to assess relationships between variables. All analyses were conducted using SPSS 26.0 (IBM, Armonk, NY, USA) with a significance level set at *P* < 0.05.

## Results

### Patient demographics and clinical data

Demographic and clinical data were collected from medical records. Among the 60 enrolled patients: Immunohistochemistry group (n = 30): 20 males, 10 females; age range: 50–88 years (mean±SD:69.23 ± 9.54); time from symptom onset to thrombectomy: 5.4 ± 2.24 hours; average hospital stay: 12.13 ± 7.28 days. Co-culture group (n = 30): 19 males, 11 females; age range: 35–84 years (mean±SD: 66.2 ± 11.47); time from symptom onset to thrombectomy: 5.36 ± 2.31 hours; average hospital stay: 10.2 ± 4.35 days (Table 1).

**Table 1:** Baseline characteristics in immunohistochemical staining and co-culture groups.

	Immunohistochemical Staining Group	Co-culture Group
Age, years, mean±SD	69.23 ± 9.54	66.2 ± 11.47
Sex, n, (male/female)	20/10	19/11
Time from Onset to Thrombectomy, h, mean±SD	5.4 ± 2.24	5.36 ± 2.31
Hospitalization Time, days, mean±SD	12.13 ± 7.28	10.2 ± 4.35

### Immunohistochemical staining of thrombi

**Fig 1:**
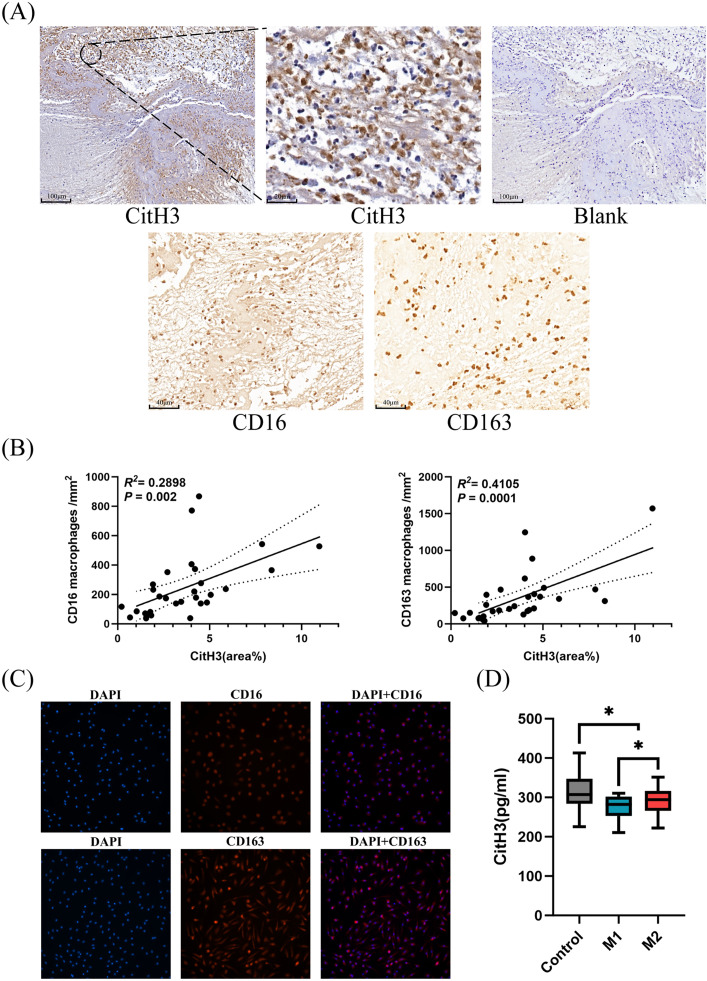
Distribution, correlation, and functional analysis of NETs and macrophages in cerebral thrombi from AIS patients. (A) Immunohistochemical staining of neutrophil extracellular traps (CitH3⁺), CD16⁺ , and CD163⁺ macrophages in cerebral thrombi. (B) Scatter plots showing the correlation between CD16⁺/CD163⁺ macrophage density and CitH3-positive area. (C) Fluorescence staining of DAPI and M1/M2 macrophages. Magnification: 100× . DAPI: 4′,6-diamidino-2-phenylindole dihydrochloride. (D) Box plots comparing CitH3 concentrations after co-culture of thrombi with M1 and M2 macrophages.

Immunohistochemical staining was performed on 30 thrombi, with the staining targets including CitH3, CD16, and CD163 (Fig 1A). After immunohistochemical staining of the 30 thrombus samples, all thrombus samples showed positive expression of CitH3, CD16, and CD163, with a positive expression rate of 100%. The thrombus area ranged from 1.716 to 10.112 mm^2^, with an average of 5.889 ± 2.502 mm^2^. The CitH3 positive expression area ranged from 0.010 to 0.726 mm^2^, with an average of 0.221 ± 0.194 mm^2^, accounting for 0.665% to 10.958% of the thrombus area. Cell counting was performed on thrombi with positive CD16 and CD163 expression, and the results showed that the number of CD16^+^ cells (n = 30) was 245.03 ± 207.84 cells/mm^2^, and the number of CD163^+^ cells (n = 30) was 347.53 ± 346.41 cells/mm^2^. Correlation analysis using the Pearson method revealed that the number of CD16^+^ and CD163^+^ cells in thrombi was positively correlated with the percentage of CitH3 positive area (Fig 1B).

### Macrophage culture and identification

The cells in the 4 wells of the culture plate were labeled. The 2 wells with the polarization target of M1 macrophages were designated as wells A and B, and the 2 wells with the polarization target of M2 macrophages were designated as wells C and D. Macrophages in well A were stained with DAPI and CD16 fluorescence. Under the fluorescence microscope, the macrophage nuclei stained with DAPI showed blue fluorescence, and the macrophages stained with CD16 showed red fluorescence. In well B, cells were stained with CD163 fluorescence, and no red fluorescence was observed, indicating that the cells in wells A and B were M1 macrophages. Macrophages in well C were stained with DAPI and CD163 fluorescence. Under the fluorescence microscope, the macrophage nuclei stained with DAPI showed blue fluorescence, and the macrophages stained with CD163 showed red fluorescence. In well D, cells were stained with CD16, and no red fluorescence was observed, indicating that the cells in wells C and D were M2 macrophages. By performing fluorescence staining on the cells cultured in parallel in the culture plate, it was demonstrated that the cell culture protocol in this study could obtain macrophages with different polarization states, and it indirectly reflected the polarization state of macrophages in the culture bottle (Fig 1C).

### Comparison of CitH3 concentration after thrombus-M1 and M2 macrophage co-culture with blank control

Before culture, the CitH3 concentration in the culture medium was 268.09 ± 35.30 pg/ml. After culture, the concentration in the blank control group was 312.39 ± 42.57 pg/ml, the concentration after co-culture of thrombus-M1 macrophages was 274.93 ± 29.04 pg/ml, and the concentration after co-culture of thrombus-M2 macrophages was 292.09 ± 33.34 pg/ml. A t-test was performed on the CitH3 concentration after co-culture, and it was found that compared with the CitH3 concentration after culture in the blank control group, the CitH3 concentration after co-culture of thrombus-M1 macrophages and thrombus-M2 macrophages was significantly lower, with statistical significance (Mean Difference (95%CI): −37.5 (−56.3,-18.6, *P* < 0.001; Mean Difference (95%CI): −20.3 (−40.1,-0.5), *P* = 0.044). And Compared with the thrombus-M2 macrophage group, the concentration of CitH3 in the culture medium after co-culture of thrombus-M1 macrophages was significantly lower (Mean Difference (95%CI): −17.2 (−33.3,-1.0), *P* = 0.038) (Fig 1D).

## Discussion

Ischemic stroke is a common cerebrovascular disease in clinical practice. Most patients still have varying degrees of neurological dysfunction after treatment, which not only reduces their quality of life but also imposes a heavy economic burden on society and families. After the occurrence of ischemic stroke, neutrophils are the first immune cells to appear in the ischemic brain tissue and produce a variety of inflammatory factors, leading to nerve damage and blood-brain barrier disruption [15].

NETs were first discovered by Volker Brinkmann and his colleagues, who pointed out in their research that NETs are capable of degrading virulence factors and eliminating bacteria, representing a new way for neutrophils to combat pathogens [3]. NETs are mainly composed of double – stranded DNA, histones, and granule proteins, with the latter including neutrophil elastase (NE), cathepsin G, and myeloperoxidase (MPO), among others [16]. CitH3 serves as a biomarker for NETs and is commonly used to detect the presence of NETs in plasma and thrombi [10]. Through immunohistochemical staining for CitH3 in 30 collected cerebral thrombus samples, CitH3-positive expression was found in all thrombi, indicating that NETs are one of the important components of cerebral thrombi, which is basically consistent with previous research results [17]. In recent years, studies have shown that activated neutrophils contribute to the pathogenesis of ischemic stroke by releasing NETs. However, few studies have investigated how macrophage subtypes differentially interact with and degrade NETs within human cerebral thrombi. Our study provides novel in situ evidence linking NET content to both M1 and M2 macrophage densities in thrombi and experimentally compares their NET-degrading capacities using patient-derived macrophages.

Common specific markers for M1 macrophages include CD16, CD32, CD80, CD86, iNOS, and MCH-II, etc. To explore the existence of M2 macrophages in thrombi, immunohistochemical staining for markers such as CD80, CD86, MCH-II, and CD16 was performed on thrombus sections. The results showed that only CD16 had positive expression in cerebral thrombus sections, while the other markers were all negative in immunohistochemical staining. Regarding the phenomenon that only CD16 was positively expressed and the rest were negative in thrombus sections, we speculate that it is related to the difference in the expression time of surface markers of macrophages during the onset of AIS. In a study inducing ischemic stroke in mice, it was found that microglial cells could detect the expression of CD16 on the first day of cerebral infarction, and it continued to increase until day 14 [18], while other markers usually began to express on the third day [19]. Since the thrombi studied in this experiment were formed after the rupture of atherosclerotic plaques and were all removed within 24 hours after the onset of the disease, it is speculated that other macrophage markers except CD16 had not yet been expressed, hence the negative results in immunohistochemical staining. However, surface markers alone cannot clearly distinguish between microglial cells and macrophages in the blood, so further research is needed to explore whether there are differences in the polarization mechanisms of macrophages in thrombi and microglial cells in the brain parenchyma.

After statistical analysis of the number of CD16^+^ and CD163^+^ cells per unit area in thrombi and the positive area of CitH3, it was found that the number of CD16^+^ and CD163^+^ cells per unit area was positively correlated with the positive area of CitH3. That is, when there were more NETs in the thrombus, the number of M1 and M2 macrophages was also higher. And from the existing sample data, the number of M2 macrophages in the thrombus was generally more than that of M1 macrophages. The occurrence of the above situation may be related to the following mechanisms: 1. NETs, as a pro – inflammatory medium, can recruit monocytes from the blood to the site of thrombus formation and further differentiate them into macrophages to clear inflammation and promote tissue repair [20–22]; 2. The main component of NETs is deoxyribonucleic acid (DNA), and its degradation and clearance require the participation of deoxyribonuclease (DNase). Macrophages can secrete a variety of DNases to degrade NETs in thrombi and absorb the remaining extracellular DNA through endocytosis [23,24]. Therefore, macrophages are important inflammatory cells for the degradation and clearance of NETs; 3. NETs can promote the polarization of monocytes into M2 macrophages, which may be the potential mechanism for the higher number of M2 macrophages than M1 macrophages in thrombi. Initially, Consol Farrera and others found that human macrophages derived from monocytes could phagocytize NETs in a cytochalasin D-dependent manner, and the phagocytosis process alone would not lead to more inflammation [12]. Subsequently, Patrick Haider and others further proved through in-vitro experiments that macrophages could secrete DNase to decompose and clear NETs and absorb the remaining extracellular DNA through endocytosis. They also pointed out that compared with M0 (undifferentiated) and M2 (anti-inflammatory) macrophages, M1 (pro-inflammatory) macrophages had a more significant effect on degrading NETs [23]. At present, there is no research to clearly explain the phenomenon that M2 macrophages are widely more than M1 macrophages in cerebral thrombi. However, in some fields of ischemic stroke, related studies have shown that NETs can promote the polarization of monocytes into M2 macrophages. A study on the specific role and mechanism of substance P in epidural fibrosis found that substance P promoted the polarization of M2 macrophages in epidural fibrosis through sphingomyelin synthase 2 and NETs [25]. Guimarães-Costa et al. found in the study of Leishmania that NETs could reprogram the differentiation of monocytes induced by IL-4/GM-CSF into M2 macrophages [26].

Previous studies on the ability of macrophages to clear NETs were mostly based on healthy individuals. In order to explore the ability of macrophages derived from monocytes in ischemic stroke patients to clear NETs, we designed the subsequent co-culture control experiment. Through the co – culture experiment, it was found that compared with the blank control group, the concentration of CitH3 in the culture medium after co-culture of thrombus-M1 macrophages and thrombus-M2 macrophages was lower, indicating that M1 and M2 macrophages derived from human monocytes both had the ability to degrade and clear NETs. Moreover, through statistical analysis of the CitH3 concentration in the culture medium after M1 macrophage group and M2 macrophage group, it was found that the CitH3 concentration in the culture medium after co-culture of thrombus - M1 macrophages was lower than that of M2 macrophages. Therefore, compared with M2 macrophages, M1 macrophages may have a stronger ability to degrade and clear NETs in thrombi, which is basically consistent with the previous research results [23]. Combining the findings of this study, in clinical practice, the difference in the ability of macrophages with different polarization states to clear NETs can be utilized. Through drugs or other therapeutic means, monocytes can be induced to polarize more towards M1 macrophages during the process of thrombus formation and subsequent inflammatory response in ischemic stroke, accelerating the degradation and clearance of NETs, promoting thrombolysis and vascular recanalization, and at the same time reducing the secondary nerve damage caused by NETs. This can provide a new, safer, and more effective treatment method for clinical treatment of ischemic stroke and try to improve the prognosis of patients as much as possible.

This study has several limitations that should be acknowledged. First, although M1 macrophages exhibited stronger NET-degrading capacity in vitro, their pro-inflammatory properties raise concerns about potential neurotoxicity or exacerbation of inflammation in vivo. Therefore, therapeutic strategies aimed at promoting M1 polarization must be pursued with caution and require further mechanistic and translational validation. Second, the in vitro co-culture model employed in this study may not fully replicate the complex cellular and molecular milieu of the ischemic brain, which involves dynamic interactions among endothelial cells, platelets, cytokines, and other immune populations. Third, NET degradation was assessed solely by measuring CitH3 levels, a specific but limited marker of NETs. Incorporating additional markers—such as extracellular DNA, neutrophil elastase (NE), or employing direct imaging techniques—would provide a more comprehensive evaluation of NET clearance. Lastly, the relatively small sample size (30 patients per group) may limit the statistical power and generalizability of our findings. Future studies with larger, multicenter cohorts are warranted to validate these observations.

## Conclusion

In summary, this study demonstrated the presence of NETs in cerebral thrombi and the correlation between macrophage polarization and NET content. Both M1 and M2 macrophages were found to have the capacity to degrade and clear NETs, with M1 macrophages exhibiting a stronger effect.

## Supporting information

S1 File Patient info. Contains anonymized demographic and clinical information for all AIS patients.
(XLSX)

S2 File Thrombus Cit and macrophages. Morphological measurements of thrombus CitH3-positive area and macrophage densities.
(XLSX)

S3 File CitH3 culture. ELISA-based quantification of CitH3 concentrations before and after co-culture.
(XLSX)
